# Frost Work

**Published:** 1878-03

**Authors:** 


### FROST WORK.'

There is beauty in a splendid education, where the mind has been trained in all the accuracy of mathematics, in all the elevating elegances of poetry, and painting, and sculpture, and music. And grammar, and logic, and rhetoric may have been thoroughly mastered and reduced to the practice of an accomplished writer and a finished orator, an abstract morality may pervade every line written, every uttered sentiment. But who does not know, that these things alone, never make a loveable character, there is beauty in it, as cold as the icicle, as dead as the marble, as transient as the clouds of morning, the body is without warmth, the heart without sympathy, the whole nature without love, beyond the circle of its own cold and dead, and magnificent self.

The son of a London Alderman, was William Beckford, left fatherless at ten, with an annual income of half a million of dollars. No pains were spared to give him a refined education. The great Mozart taught him music. Sir William Chambers gave him lessons in architecture. At the moment of his majority, he launched out upon the world, proud, haughty and accomplished, withdrew from men, encircled his domains with a two mile wall, razeed his father's residence, which had been built at an expense of more than a million of dollars, and erected another on its site, in mid-winter, men working day and night, and amid gorgeous palaces, in more than eastern magnificence, he passed his time in luxurious ease. He feasted in the contemplation of his own grandeur, but the day was not long enough for that, for at times when his whole establishment was lighted up with torches at mid-night, he would repair to a distant elevation, and gaze upon it by the hour. All this was the result, the very natural result of an "accomplished education." Had he been brought up in another school which would have invited out the affections, which would have trained him in the exercise of a benevolent nature; had he been been taught that the best way to happyfy himself was to be employed in happyfying others, he would most probably have lived to high purposes, and would have gone down to the grave with the benedictions of multitudes on his head, to be repeated as to his memory, by every successive generation, for all time. Instead of which his towers fell with their own weight, property depreciated, law suits terminated adversely, the sheriff entered where royalty had been refused, and he fell unpitied. Font Hill Abbey, like its owner, was levelled to the earth, and the wealth and the name of the author of *Vathek*, have faded away "like frost work before the sun." From this narration, many of the families of this land may derive instruction. The great aim of multitudes of parents, especially in cities, is to afford their daughters a splendid education, and the music of harps and guitars and pianos, is assiduously cultivated for months and years, instead of the infinitely more purifying and harmonizing "psalms and hymns, and spiritual songs" of Isaac Watts. The obscene and impossible mythology of classical poets is studied more than the real truths of scripture history, and the dance, and the ball, and the opera, are patronized before the prayer meeting, the lecture, and the sabbath worship. The result of all this is, our daughters grow up dressy, idle, proud, vain and worthless as to the real and necessary duties of life. They live in sham and show, and a vain parade, of which they soon get weary, because of their unsatisfying nature; life has no absorbing object; that of it which they see is tame and tasteless, there is nothing in it to stimulate them to energizing activities of mind or heart or body, and they pine away in listlessness and early disease, or, to protract for a brief space the flickering lamp of life, they feed it with opiates, or fill it with alcohol.

